# Spent Yeast Valorization for Food Applications: Effect of Different Extraction Methodologies

**DOI:** 10.3390/foods11244002

**Published:** 2022-12-10

**Authors:** Ana Sofia Oliveira, Joana Odila Pereira, Carlos Ferreira, Margarida Faustino, Joana Durão, Ana Margarida Pereira, Carla Maria Oliveira, Manuela E. Pintado, Ana P. Carvalho

**Affiliations:** 1 Escola Superior de Biotecnologia, CBQF—Centro de Biotecnologia e Química Fina—Laboratório Associado, Universidade Católica Portuguesa, Rua Diogo Botelho 1327, 4169-005 Porto, Portugal; 2 Amyris Bio Products Portugal Unipessoal Lda, Rua Diogo Botelho 1327, 4169-005 Porto, Portugal

**Keywords:** protein, engineered yeast, autolysis, high-pressure homogenization, enzymatic hydrolysis, sonication, circular economy, sustainable process, *Saccharomyces cerevisiae*

## Abstract

Over the years, synthetic biology has been growing with the use of engineered yeast strains for the production of sustainable ingredients to meet global healthcare, agriculture, manufacturing and environmental challenges. However, as seen from the brewing industry perspective, these processes generate a substantial amount of spent yeast that contains high nutritional value related to its high protein content, showing its potential to be used as an alternative protein source. Taking into account the rising demand for protein because of the growth in the global population, the present study aims to produce peptide-rich extracts by different potentially scalable and sustainable methodologies in a circular economy approach for the food and nutraceutical industries. The results demonstrated that extraction from genetically modified strains allowed the production of extracts with an excellent nutritional profile and low molecular weight peptides. Furthermore, autolysis was shown to be a potential sustainable approach for this production, though other green metrics need to be explored in order to establish this process at an industrial level.

## 1. Introduction

Protein is an essential element in every healthy diet [1], and rising demand for protein-rich foods and beverages is driving the growth of the protein food market, which is expected to increase at a compound annual growth rate (CAGR) of 7% until 2024. In fact, it has been estimated that, due to growth in population and living standards, protein consumption and demand may increase by 30 to 40% in the short term (in the next decade or so) [2]. In addition, the pharmaceutical industry is increasingly aware of the bioactive properties of protein and peptides, further increasing the demand for new sources of these biomolecules [3]. This has driven the peptide therapeutics market valorization at a CAGR of 7.9% between 2019 and 2027 [4]. Therefore, protein and peptide markets represent an opportunity ready to be explored. A recent analysis of the global supply of protein shows that vegetal sources have the highest impact (59%), together with considerable contributions from animal sources (35%) [5]. However, pressure on limited global resources, namely limited arable land, drives the need for alternative sources of protein in order to feed the ever-increasing world population with a healthy and balanced diet. Spent brewer’s yeast (SBY) is a promising alternative since it has a high protein content (up to 60% of its dry weight content [6]), it is a “Generally Recognized as Safe” (GRAS) microorganism [5] and is readily available in large quantities thanks to a growing brewing industry [7]. Moreover, the use of by-products from other processes is an integral practice in the circular economy approach, since it adds value to materials that would otherwise be discarded and relieves pressure from virgin natural resources. All of these qualities make the use of SBY an attractive alternative for the extraction of protein and peptides for the food, feed and nutraceutical industries [8]. 

While wild *Saccharomyces cerevisiae* (*S. cerevisiae*) strains have long been used in the food industry (e.g., for brewing, and bread and wine making), new genetically engineered strains have been created for tailored needs, for example, for the production of specific target molecules such as farnesene [9], propanol and butanol [10], among others [11]. Since these strains are genetically modified with the aim of improving or enabling their production capacity, alterations to genes related to structural components have been widely described, whereas only a few publications have focused on perturbation of the cell wall in metabolic engineering [12]. For that reason, many questions remain regarding the effect of changes in yeast morphology, and what effect those changes might have on extraction processes. On the one hand, cell wall structural changes may influence the capacity and yield of physical extraction methods, making the cell wall stronger or weaker, since protein release is directly dependent on its efficient breakage. On the other hand, cytosol enzymatic composition changes may interfere with chemical and enzymatic methods by increasing, for example, the resistance against the action of a particular enzyme that might be used to disrupt the cell during extraction [5]. To the best of our knowledge, there are as yet no studies into engineered yeast alteration and protein extraction.

Concerning the increase in worldwide protein demand and the amount of spent yeast generated by the brewing and synthetic biology industries, the aim of the present work is to produce peptide-rich extracts from engineered and non-engineered spent yeast by applying potentially scalable and sustainable processes at an industrial scale. This will in turn present more viable options for mass exploration of spent yeast in food and nutraceutical industries using economic and sustainable approaches. As for *S. cerevisiae* extraction methods, thorough reviews have already been conducted on the different physical, chemical and enzymatic methods generally used for SBY protein extraction [5]. From the described methods generally used for SBY protein extraction, physical methodologies (high-pressure homogenization (HPH) and sonication), autolysis and enzymatic hydrolysis (enzymatic methods) have been selected, thus covering the majority of the processes used. 

To meet the objectives noted above, two steps were taken. First, the effectiveness of these methods in extracting protein and peptides from spent yeast was evaluated using a range of parameters such as protein content of extracts produced and their molecular weight (MW) distribution. Second, the processes for protein recovery and sustainable metrics were calculated in order to study the application of the extraction process at an industrial level. These parameters were also used to assess the possible differences that distinct engineered strains may or may not place on the extraction processes. 

## 2. Materials and Methods

### 2.1. Yeast Strains

Spent yeast of two different *S. cerevisiae* strains, namely “ESY1” and “ESY2”, engineered by Amyris, Inc. to produce β-farnesene were used in this study. A non-engineered spent brewer’s yeast (*Saccharomyces)* kindly supplied by Super Bock Group was used as control (“CSY”). Samples were transported under refrigerated conditions and processed immediately after collection.

### 2.2. Yeast Pre-Treatment

After collection, spent yeast was immediately subjected to two washing processes in order to remove residual components of the fermentation process (11,949× *g*, 10 min, 4 °C). The supernatant was discarded and the washed yeast pellet was distributed for the different extraction methods. A sample of spent yeast and washed yeast pellet was collected to determine dry weight and protein content. 

### 2.3. Extraction Methods

Two physical and enzymatic methodologies described in the literature were used for protein and peptides extraction from *S. cerevisiae* [5]. The cell suspension for all methods were prepared according to wet yeast weight.

#### 2.3.1. High Pressure Homogenization (HPH)

HPH was conducted based on Ekpeni et al. [13] with some modifications. A cell suspension of 200 mL at 39% (*w*/*v*) was prepared in deionized water. Five continuous passages were performed in GEA Lab Homogenizer PandaPLUS 2000 (GEA Group AG, Düsseldorf, Germany), with pressure varying from 600 to 1000 bar during the process since the yeast cell suspension viscosity was increasing. The samples were collected in an ice bath to avoid overheating. At the end of HPH, the cell suspension was centrifuged (15,777× *g*, 10 min, 4 °C). 

#### 2.3.2. Sonication

The protocol of sonication was adapted from Liu et al. [14] using a JP Selecta CY-500 Ultrasonic Homogenizer (500 W, 20 kHz) coupled with a cylindrical titanium alloy probe tip (Ø 10 mm) (Barcelona, Spain). A total volume of 200 mL of 1% cell suspension (*w*/*v*) in deionized water was irradiated at 60% amplitude for 30 min in an ice bath (pulse durations of 60 s on and 15 s off). At the end of sonication, the cell suspension was centrifuged (15,777× *g*, 10 min, 4 °C). 

#### 2.3.3. Autolysis

A methodology from Jacob et al. [15] was used to autolyze yeast with some modifications. The washed yeast pellet was dissolved in deionized water (proportion of 1:1(*w*/*v*)) and the autolysis was performed during 16 h at 50 °C with continuous stirring (120 rpm) using a New Brunswick™ Innova^®^ 40/40R Benchtop Orbital Shaker (Eppendorf, Hamburg, Germany). After 16 h, the intrinsic enzymes were inactivated at 95 °C during 5 min. At the end, the cell suspension was centrifuged (4696× *g*, 10 min, 4 °C). 

#### 2.3.4. Enzymatic Hydrolysis

Enzymatic hydrolysis was carried out using the Chae et al. [16] method. A 20% cell suspension (*w*/*v*) was prepared in phosphate buffer 100 mM pH 6.5 (sodium phosphate monobasic, Sigma-Aldrich, Inc., St. Louis, MO, USA) in order to maintain the optimum pH for enzyme activity. Enzymatic cocktails were added to the sample: 2.0% of Flavourzyme^®^, protease from *Aspergillus oryzae* 500 LAPU/g, and 0.6% Protamex^®^, protease from *Bacillus* sp. 1.5 AU-N/g (Sigma-Aldrich, Inc., St. Louis, MO, USA). The amounts were calculated according to the protein content of spent yeast. The hydrolysis was conducted at 50 °C for 12 h with continuous stirring (120 rpm) using a New Brunswick™ Innova^®^ 40/40R Benchtop Orbital Shaker (Eppendorf, Hamburg, Germany). After 12 h, the enzymes were inactivated at 95 °C for 5 min. At the end, the cell suspension was centrifuged (4696× *g*, 10 min, 4 °C). 

At the end of each extraction process, supernatants rich in protein and peptides were collected and freeze-dried (Freeze-dryer Alpha 2–4 LSCbasic, Martin Christ, Osterode am Harz, Germany). 

### 2.4. Sustainable Metrics

As a further criterion to evaluate the extraction processes employed, different metrics of green chemistry and sustainability were calculated for each process. The metrics were calculated as described by Sheldon et al. [17]. The process pass intensity (PMI), water intensity (WI) and energy intensity score (ESI) were calculated according to the following formulae:

PMI = total mass in process/mass of final product (1)

WI = mass of water in process/mass of final product (2)

ESI = energy consumption per batch mass of final product (3)

### 2.5. Yeast Extract Characterization

#### 2.5.1. Protein

Protein content of extracts was determined according to Dumas et al. [18] by using a Dumatec™ 8000 (Foss, Hilleroed, Denmark). Approximately 50 mg of sample was weighed for aluminium crucible and the analysis was performed at helium and oxygen flow rates of 195 mL/min and 300 mL/min, respectively, at 1100 mbar. The protein content was determined from the total nitrogen (N_2_) content, multiplied by a conversion factor of 5.5 because of the high content of non-protein nitrogen in yeast [19]. A calibration curve from 10 mg to 150 mg of EDTA calibration standard (Foss, Hilleroed, Denmark) was used for protein calculation. 

#### 2.5.2. Dry Weight

Dry weight was determined at 105 °C for 24 h according to standard procedures of the Association of Official Analytical Chemists (AOAC, 2005).

#### 2.5.3. Protein and Peptides MW

The protein and peptide MW distribution of yeast extracts was analyzed based on LC−ESI−UHR−QqTOF−MS method of Oliveira et al. [20]. An ultra-high-performance liquid chromatography system from Bruker Elute series, coupled to an ultrahigh-resolution quadrupole−quadrupole time-of-flight (UHR−QqTOF) mass spectrometer (Impact II; Bruke Daltonik GmbH, Bremen, Germany) with an Intensity Solo 2 C18 (100 × 2.1 mm, 2.2 μm, Bruker Daltonik GmbH, Bremen, Germany) (BRHSC18022100) was used to analyze the protein and peptide MW distribution of yeast extracts at MS positive mode (150 to 2200 *m*/*z*). Water with 0.1% of formic acid (A) and acetonitrile with 0.1% of formic acid (B) were used as mobile phases at 0.250 mL/min flow rate in gradient mode. ESI-L Low Concentration Tuning Mix (Agilent Technologies Inc., Santa Clara, CA, USA) was used for post-acquisition internal mass calibration at each analysis.

#### 2.5.4. Amino Acids

The amino acids quantification was assessed as described by Long et al. [21] by ortho-phthalaldehyde (OPA)-derivatization using a Chromolith^®^ Performance RP18 (4.6 × 100 mm) column (Merck KGaA, Darmstadt, Germany) for separation by reverse-phase high- performance liquid chromatography coupled to a high-resolution fluorescence detector (Agilent Technologies, Inc., Santa Clara, CA, USA). Before HPLC analysis of total amino acids, an acid hydrolysis (20 h, 115 °C) was performed at 10 mg of extract in 3 mL HCl 6 M under anoxic conditions. Then, pH was adjusted to 3.2 and the solution diluted to a final volume of 10 mL [22]. The derivatization of 20 μL of extract was done in automatic liquid multisampler and 10 μL was injected.

Analysis was done in triplicate and the amino acids quantified according to calibration curves of pure standards (Sigma-Aldrich, Inc., St. Louis, MO, USA) from 1 to 30 mg/L, using norvaline (Sigma-Aldrich, Inc., St. Louis, MO, USA) as internal standard.

#### 2.5.5. Neutral Sugars

Neutral sugars derivatized to their alditol acetates were analyzed by gas-chromatography-flame ionization detection as described by Pinto et al. [23] in a 7890B GC System with a DB-225 capillary column (30 m length, 0.25 mm diameter, 0.15 µm thickness) (Agilent Technologies, Inc., Santa Clara, CA, USA). Before the derivatization reaction, the extracts were hydrolysed using 72% H_2_SO_4_ (Honeywell, NC, USA) (3 h, room temperature) followed by 1M H_2_SO_4_ (2.5 h, 100 °C) [24]. The released monosaccharides were converted to their respective alditol acetates using the protocol of Blakeney et al. [25]. Two microliters of sample were injected into the GC–FID and the analysis was performed at split mode with a ratio of 1:60 (200 °C). The initial oven temperature was set for 200 °C and kept for 2 min, then elevated at the rate of 40 °C/min to 220 °C (7 min hold) followed by increase to 230 °C at 20 °C/min (5 min hold) with the total time of analysis being 15 min. The carrier gas was nitrogen at a constant flow of 1 mL/min. The equipment was coupled to a hydrogen generator (30 mL/min), using compressed air at 400 mL/min during the analysis. 

#### 2.5.6. Minerals

An optical emission spectrometer Model Optima 7000 DV ™ ICP-OES (Dual View, PerkinElmer Life and Analytical Sciences, Shelton, CT, USA) with radial configuration was used for minerals analysis following the procedure of Chatelain et al. [26]. A calibration curve of commercial mix standards for ICP analysis (Inorganic Ventures, Christiansburg, Christiansburg, VA, USA) (molybdenum, zinc, cadmium, phosphorus, lead, nickel, cobalt, boron, manganese, iron, magnesium, calcium, copper, aluminium, sodium and potassium) from 0.05 to 10 mg/L was applied for quantification. A microwave digestion was performed before ICP analysis in a Speedwave XPERT (Berghof Products + Instruments GmbH, Eningen, Germany) using 250 mg of sample with 6 mL of Suprapur^®^ HNO_3_ and 1 mL of 35% H_2_O_2_ (Merck KGaA, Darmstadt, Germany).

### 2.6. Statistical Analysis

Statistical analysis was performed using Real Statistics Resource Pack software (Release 7.2) and the results expressed as mean ± standard deviation from assay replicates. Two different fermentation reactors were collected for each spent yeast and each extraction method was performed in triplicate. Procedures of extracts characterization were assessed in triplicate.

Normality of data was tested using the Shapiro-Wilk’s test and the comparison between different extraction methods and/or yeasts was performed using a one-way ANOVA followed by Tukey’s multiple comparisons test, after evaluating the homogeneity of variances using the Levene Test for Equality of Variances. 

## 3. Results and Discussion

### 3.1. Peptide-Rich Extracts Characterization

#### 3.1.1. Protein Content (% *w*/*w*)

All the extraction methodologies applied to engineered and non-engineered spent yeast allowed the production of extracts that can be labeled as “rich in protein” [27], since a range of protein content from 25.3% to 64.8% (*w*/*w*) was observed (Table 1). These results were in accordance to protein content obtained by Ganeva et al. [28], Podpora et al. [29] and Ferreira et al. [30]. Indeed, the initial protein amount of the raw strains showed already high values from a nutritional point of view (32.9 to 43.0%), highlighting the potential use of engineered spent yeast for mass exploration in food and nutraceutical industries such as non-engineered yeasts. However, the increase in protein content in the final product was still a challenge and, for that reason, different processes were optimized [5].

An increase in protein content in extracts was observed in relation to raw yeast, after applying the extraction methodologies with the exception of the hydrolysis process (Table 1, Tables S1 and S2). Enzymatic hydrolysis is based on the application of exogenous enzymes to break the yeast cell wall at specific sites to release its protein and peptides. In general, it has been explored in food and nutraceutical industries for the production of yeast hydrolysates, using commercial proteolytic enzymes, which are responsible for the composition of peptides produced [5]. For this reason, several studies have been performed in order to assess the best hydrolysis conditions for maximum enzyme efficiency, such as enzyme dosage, pH, time, and temperature, to be applied to a specific yeast [16,31,32]. In the present study, hydrolysis conditions optimized by Chae et al. [16] were used, since they obtained a high protein yield using *S. cerevisiae* in comparison with other studies [5]. However, these conditions were not optimized for the specific strains used here, being the potential reason for observing the decrease in protein content in extracts in comparison with raw yeasts. For all yeasts, autolysis, sonication and HPH were the best processes for rich protein extract production (Table 1, Tables S1 and S2). As reviewed by Oliveira et al. [5], autolysis is more advantageous than enzymatic hydrolysis and physical methods as it is based only on the action of the yeast’s own enzymes, when cell death phase is initiated, which leads to degradation of cell components and consequently the release of protein and peptides. Unlike sonication energy-related consumption, operational and economical limitations, HPH is widely accepted at the industrial scale. However, HPH equipment requires regular and costly maintenance since they easily become clogged.

Regarding the comparison between the strains, no tendencies were observed since the differences in the protein content of extracts seemed to be more affected by the extraction method than by the type of strain used (Table 1, Tables S1 and S2). 

#### 3.1.2. MW Distribution

**Figure 1:**
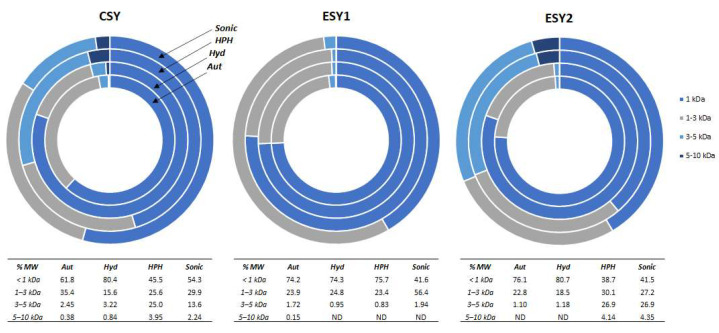
Peptides molecular weight distribution (% MW) in peptide-rich extracts obtained from each extraction process (Aut—autolysis, Hyd—enzymatic hydrolysis, HPH—high pressure homogenization, Sonic—sonication) applied to the different yeast strains (CSY, ESY1 and ESY2). ND—not detected (below low detection limit).

MW distribution of protein and peptides is a standard analysis for the evaluation of spent yeast extraction methods, since different methodologies result in the release of molecules with different sizes, according to their operation mode and extension [5]. Different MW profiles were obtained between yeast strains and extraction processes, as shown in Figure 1.

For the three yeast strains, the percentage of peptides under 1 kDa was highlighted for all extractions, in agreement with Oliveira et al. [8], where there was found a range from 38.7 to 80.7%. In fact, MW has an important role on peptides bioactivity since those of small size (3 to 20 amino acids) are described as highly specific in their biological function and choice of biological target [3]. However, some peptides with a size of 1–3 kDa were also described by their antioxidant- and angiotensin-converting enzyme (ACE) inhibitory activities [33,34]. In fact, a percentage from 15.6 to 56.4% of 1–3 kDa peptides was also observed in this study, suggesting the potential bioactivities of our extracts. On the other hand, lower percentages of peptides of about 3–5 kDa were found, followed by residual amounts of 5–10 kDa peptides. 

Concerning peptides < 1 kDa of different strains, no alterations were observed between strains, with the exception of ESY1 HPH (75.7%) that stands out from CSY (45.5%) and ESY2 (38.7%). Regarding the other potential bioactivity fraction (1–3 kDa), the ESY1 sonication (56.4%) pointed out in relation to CSY (29.9%) and ESY2 (27.2%). However, as demonstrated earlier in the protein content results, no differences were noted between genetically modified and control strains (Figure 1). 

Looking at the global results of three yeasts, enzymatic methods seemed to allow a high percentage of bioactive peptides under 3 kDa (CSY: ~97%, ESY1: ~99%, ESY2: ~99%). Indeed, autolysis process has been generally described for releasing oligopeptides (2–3 kDa) followed by di-, tri-, and tetra-peptides (<600 Da) but the size of released peptides is always dependent on controlling the autolysis extension, since peptides of different MW can be obtained on the same process because of their degradation during lysis process [35]. Our results are similar to those obtained by Jacob et al. [15] since they observed an increase in peptides < 4 kDa and free amino acids at enzymatic protocols in comparison with bead milling and ultrasound. 

### 3.2. Extraction Methodology Evaluation

As reviewed by Oliveira et al. [5], several physical, chemical and enzymatic methodologies are available for extracting protein and peptides from spent yeast, and a combination of methods is common practice at the industrial level. However, the selection of methods is directly related to the intended application of the final product for maximum protein recovery and quality [7]. Protein isolation and purification protocols are often coupled to these extractions in order to fulfill the needs of a specific economic sector [5]. That having been said, the food and nutraceutical industries have been facing a challenge in establishing a viable process at economic and sustainable levels, since several of the technologies cannot easily be made efficient, reproducible, low-cost and scalable. In this segment, the protein recovery of each process applied at different yeast strains and the respective sustainable metrics were evaluated. 

#### 3.2.1. Protein Recovery 

**Figure 2:**
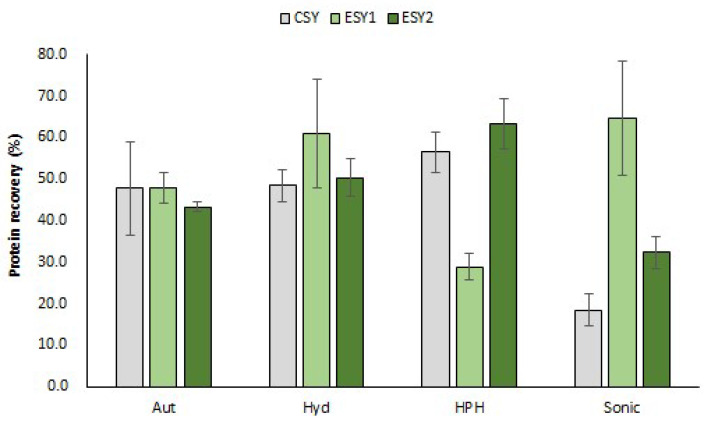
Protein recovery (%) of each extraction process (Aut—autolysis, Hyd—enzymatic hydrolyis, HPH—high pressure homogenization, Sonic—sonication) applied to the different yeast strains (CSY, ESY1 and ESY2).

From all processes, the percentage of protein recovery was calculated based on the protein amount of raw yeast in relation to the protein of produced extracts. As observed in Figure 2, protein recovery from 18.5 to 64.9% was obtained from all processes. Indeed, several variables influence protein recovery values, such as temperature, processed volumes, cell suspension concentration, electric field strength and time of treatment, and it is quite difficult to establish the best process [5].

ESY1 sonication showed the highest protein recovery (64.9 ± 13.9%) from all processes. On the other hand, a statistically significant low protein recovery was verified for the other engineered yeast (ESY2: 32.4 ± 3.8%) in comparison with ESY1 on the same extraction methodology, despite both being higher than control yeast (18.5 ± 3.8%) (Figure 2; Tables S3 and S4). Indeed, this result corroborates the abovementioned hypothesis that the extraction method may have more influence at protein obtained than the type of yeast strain used. A similar result was obtained for other processes applied, since there were statistically significant (*p* < 0.05) differences between ESY1 and ESY2 at HPH (28.9 ± 3.3% and 63.5 ± 6.0%, respectively), confirming the abovementioned assumption (Figure 2; Tables S3 and S4). Regarding the comparison between extraction processes inside each strain, no trend was found, since enzymatic processes and HPH allowed the highest protein recovery for CSY and ESY2, the opposite to ESY1 which featured sonication together with enzymatic methodologies. Therefore, it was not possible to select the best method for spent yeast protein extraction based on protein recovery because of the lack of reproducibility between yeast strains. 

#### 3.2.2. Sustainable Metrics

Since spent yeast is used for the production of potential animal protein alternatives such as protein-rich extracts in a circular economy approach to fermentation industrial processes [8], it appears important to establish a more sustainable and greener process for production in the food and nutraceutical sectors. For this reason, sustainable metrics were calculated for each process conducted process in order to measure their comparative “greenness” (Table 2). 

From the metrics of all processes, there seem to be no differences among the three yeast strains. However, concerning the processes, autolysis showed low values of PMI and WI, followed by HPH, enzymatic hydrolysis and sonication, which means autolysis requires the lowest amount of raw yeast and water volume for the production of protein-rich extracts. In fact, autolysis has been described as an environmentally sustainable methodology for the recovery of intracellular compounds [36]. On the other hand, evaluating the EIS, all the processes showed extremely high metrics, an output related to the use of laboratory scale equipment, which does not allow us to make an accurate assessment in terms of energy consumption. However, from the four extractions, HPH seems to have the lowest energy consumption. 

### 3.3. Choice of Peptide-Rich Extract for Future Purification Process

After the characterization of peptide-rich extracts produced in terms of protein content and MW, and evaluation of protein recovery and sustainability of processes tested, the extract obtained from yeast strain ESY1 by autolysis was chosen for future work related to the isolation and purification of engineered spent yeast peptides. The choice was based on protein content: 54.5 ± 1.0% for ESY1 versus 48.3 ± 1.0 for ESY2 (Table 1). Furthermore, there was no observed tendency of differences between engineered and non-engineered strains, such as protein content, MW distribution and protein recovery, which enhance the potential of use of these genetically modified strains for food applications. In fact, results suggest that some of the differences observed in nutritional performance could be more related to the extraction process per se rather than the type of yeast strain used for the process. Regarding the extraction process, autolysis was revealed to be a highly efficient and sustainable scalable process. It allowed a high protein content of final extracts on engineered strains (Table 1) with a large percentage of peptides under 3 kDa (~98%) (Figure 1), which may be potentially bioactive for the human body as extensively described in the literature [3]. In addition, this process required the lowest amount of biomass for extract production, with the smallest water consumption (Table 2). These represent the first steps in establishing a green and sustainable process in the food and nutritional sectors. However, a deep evaluation of autolysis energy consumption needs to be performed at the industrial level. 

Beyond the protein-related parameters evaluated, a more complete nutritional characterization of ESY1 autolysis extract is represented in Table 3 regarding its potential application in the food and nutraceutical sectors. 

The protein content of ESY1 autolysate was shown to be higher than those obtained by Bertolo et al. [37] (39.3 ± 0.9%) and Jacob et al. [38] (42.4 ± 3.0%). Several studies described high protein percentages in autolysates, but they used purification processes coupled with extraction in order to increase the content of the protein final product [8,39], which raises interest in future work related to peptide purification processes. Similarly, the amount of sugars and minerals obtained was according to the range reviewed by Marson et al. [7]. Furthermore, the total and individual amounts of essential amino acids (EAA) exceeded Food Agriculture Organization (FAO) and World Health Organization (WHO) recommendations (Table 4) (with exception of methionine) which highlights their potential use in the food and nutraceutical industries. Indeed, these organizations describe spent brewer’s yeast as a potential source of EAA [40], a recommendation corroborated by the present study. The obtained results are according to the amino acids range of autolysates obtained by Podpora et al. [35] and Jacob et al. [15].

## 4. Conclusions

The present study showed that engineered modified spent yeast has the same performance in terms of peptide-rich extracts production than the non-engineered type, being an alternative to animal protein sources by the food and nutraceutical industries. Indeed, the nutritional analysis of extracts obtained showed their potential to be applied for a healthy and balanced diet. Furthermore, the use of a significant amount of spent yeast generated by fermentation industries offers an important opportunity to establish a circular economy in the industry. When pursuing a sustainable and green approach to producing ingredients, autolysis was the extraction methodology selected due to its interesting sustainable metrics in terms of biomass and water consumption, and high protein content obtained, as well as low MW peptides. However, an energy assessment of this process and a respective economic balance at an industrial level are paramount in order to evaluate its effective greenness and sustainability. Further studies about the isolation of peptide fractions and/or specific peptides of ESY1 autolysate need to be performed in order to evaluate potential bioactivities.

## Figures and Tables

**Table 1:** Protein content (% *w*/*w*) of raw yeast and extracts obtained from each extraction process applied to different yeast strains (CSY, ESY1 and ESY2).

	CSY	ESY1	ESY2
Raw yeast	43.0 ± 1.1	38.4 ± 2.3	32.9 ± 0.4
Autolysis	64.8 ± 2.4	54.5 ± 1.0	48.3 ± 1.0
Enzymatic hydrolysis	34.6 ± 2.6	30.8 ± 0.4	25.3 ± 0.7
HPH	51.2 ± 3.3	45.6 ± 1.7	44.5 ± 1.8
Sonication	50.2 ± 14.6	50.1 ± 4.3	46.3 ± 0.9

**Table 2:** Sustainable metrics (PMI—Process Mass Intensity, WI—Water Intensity, EIS—Energy Intensity Score) of each extraction process applied to different yeast strains (CSY, ESY1 and ESY2).

		CSY	ESY1	ESY2
Autolysis	PMI	22	27	26
	WI	11	13	13
	EIS	5764	6891	6601
Hydrolysis	PMI	35	36	35
	WI	29	30	28
	EIS	2250	2330	2217
HPH	PMI	27	66	29
	WI	19	48	21
	EIS	44.2	110	47.2
Sonication	PMI	1904	919	1509
	WI	1882	909	1492
	EIS	2353	1137	1865

**Table 3:** Protein content (% *w*/*w*), sugars (% *w*/*w*) and minerals (ng/g extract) of peptide-rich extract obtained from autolysis of ESY1.

Protein (%)	Sugars (%)	Minerals (ng/g)				
		P	Mg	Ca	K	Total
54.5 ± 1.0	14.8 ± 0.7	14.2 ± 1.0	4.34 ± 0.37	1.55 ± 0.13	16.5 ± 0.5	36.9 ± 1.9

Results are expressed in average ± standard deviation (n = 3). ND—Not detected (below low detection limit), P—Phosphorus, Mg—Magnesium, Ca—Calcium, K—Potassium.

**Table 4:** Essential amino acids content (EAA) (mg/g protein) of peptide-rich extract obtained from autolysis of ESY1.

	EAA	FAO/WHO Reference ^c^
Cys	6.94 ± 0.41	6.0
His	47.1 ± 5.0	15.0
Thr	60.4 ± 2.4	11.0
Arg	59.1 ± 3.9	NM
Val	72.8 ± 4.4	15.0
Met	8.97 ± 1.89	16.0
Phe	43.7 ± 2.6	21.0 ^b^
Tyr ^a^	41.3 ± 3.6	
Ile	56.9 ± 3.2	15.0
Leu	78.1 ± 4.8	21.0
Lys	69.9 ± 2.5	18.0
Total	545 ± 35	138

Results are expressed in average ± standard deviation (n = 3). ^a^ Non-essential, ^b^ Phe + Tyr. ^c^ World Health Organization [41] NM—not mentioned. Cys—Cysteine, His—Histidine, Thr—Threonine, Arg—Arginine, Val—Valine, Met—Methionine, Phe—Phenylalanine, Tyr—Tyrosine, Ile—Isoleucine, Leu—Leucine, Lys—Lysine.

## Data Availability

Data is contained within the article or supplementary material.

## Supplementary Materials

The following are available online at https://www.mdpi.com/article/10.3390/foods11244002/s1, Table S1. *p*-values of comparison of protein content (% *w*/*w*) between different yeast strains. Table S2. *p*-values of comparison of protein content (% *w*/*w*) between different extraction methodologies. Table S3. *p*-values of comparison of protein recovery (%) between different yeast strains. Table S4. *p*-values of comparison of protein recovery (%) between different extraction methodologies.
