# Supplementary material for: Spent Yeast Valorization for Food Applications: Effect of Different Extraction Methodologies
Source: Foods. 2022 Dec 10;11(24):4002. doi: 10.3390/foods11244002 (PMC9777911; doi:10.3390/foods11244002)
Supplement: Supplementary file 1 [file foods-11-04002-s001.zip › foods-2015029-supplementary.pdf]

Table S1. P-values of comparison of protein content (% w/w) between different yeast strains.

	**Autolysis**	**Hydrolysis**	**HPH**	**Sonic**
**CSY vs. ESY1**	2.57×10^{-7}	3.94×10^{-3}	4.19×10^{-3}	1.00
**CSY vs. ESY2**	3.43×10^{-9}	2.15×10^{-6}	2.53×10^{-3}	7.98×10^{-1}
**ESY1 vs. ESY2**	2.06×10^{-4}	4.48×10^{-4}	7.71×10^{-1}	8.10×10^{-1}

n (CSY and ESY1) = 6, n(ESY2) = 4

Table S2. P-values of comparison of protein content (% w/w) between different extraction methodologies.

	CSY	ESY1	ESY2
**Hydrolysis vs. autolysis**	6.65×10^{-6}	8.11×10^{-13}	4.44×10^{-12}
**Hydrolysis vs. raw yeast**	6.57×10^{-1}	7.28×10^{-3}	2.96×10^{-5}
**Hydrolysis vs. HPH**	7.85×10^{-3}	5.74×10^{-9}	4.30×10^{-11}
**Hydrolysis vs. sonication**	1.31×10^{-2}	4.22×10^{-11}	1.48×10^{-11}
**Autolysis vs. raw yeast**	1.45×10^{-2}	4.88×10^{-7}	7.93×10^{-9}
**Autolysis vs. HPH**	3.67×10^{-2}	2.13×10^{-5}	3.37×10^{-3}
**Autolysis vs. sonication**	2.25×10^{-2}	3.44×10^{-2}	1.60×10^{-1}
**Raw yeast vs. HPH**	6.69×10^{-1}	1.06×10^{-2}	2.42×10^{-7}
**Raw yeast vs. sonication**	7.62×10^{-1}	5.36×10^{-5}	4.34×10^{-8}
**HPH vs. sonication**	9.99×10^{-1}	2.78×10^{-2}	2.38×10^{-1}

n (CSY and ESY1) = 6, n(ESY2) = 4

Table S3. P-values of comparison of protein recovery (%) between different yeast strains.

	Autolysis	Hydrolysis	HPH	Sonic
**CSY vs. ESY1**	1.00	6.47×10^{-2}	3.50×10^{-7}	2.23×10^{-6}
**CSY vs. ESY2**	6.32×10^{-1}	9.34×10^{-1}	9.14×10^{-2}	8.07×10^{-2}
**ESY1 vs. ESY2**	6.18×10^{-1}	1.83×10^{-1}	9.48×10^{-8}	2.88×10^{-4}

n (CSY and ESY1) = 6, n(ESY2) = 4

Table S4. P-values of comparison of protein recovery (%) between different extraction methodologies.

	CSY	ESY1	ESY2
**Hydrolysis vs. autolysis**	9.98×10^{-1}	1.32×10^{-1}	1.47×10^{-1}
**Hydrolysis vs. HPH**	1.86×10^{-1}	8.55×10^{-5}	4.85×10^{-3}
**Hydrolysis vs. sonication**	1.09×10^{-6}	9.13×10^{-1}	3.47×10^{-4}
**Autolysis vs. HPH**	1.39×10^{-1}	1.52×10^{-2}	1.23×10^{-4}
**Autolysis vs. sonication**	1.51×10^{-6}	3.65×10^{-2}	1.64×10^{-2}
**HPH vs. sonication**	2.35×10^{-8}	2.06×10^{-5}	1.45×10^{-6}

n (CSY and ESY1) = 6, n(ESY2) = 4
